# Synthesis of nickel nanoparticles by a green and convenient method as a magnetic mirror with antibacterial activities

**DOI:** 10.1038/s41598-020-69679-4

**Published:** 2020-07-28

**Authors:** Mohammad Reza Ahghari, Vahhab Soltaninejad, Ali Maleki

**Affiliations:** Catalysts and Organic Synthesis Research Laboratory, Department of Chemistry, Iran University of Science and Technology, Tehran, 16846-13114 Iran

**Keywords:** Chemical biology, Chemistry, Materials science, Nanoscience and technology, Optics and photonics

## Abstract

In this work, a simple protocol was described for the synthesis of nickel magnetic mirror nanoparticles (NMMNPs) including antibacterial activities. The identification of NMNPs was carried out by field-emission scanning electron microscopy (FESEM) images, energy-dispersive X-ray (EDX) analysis, X-ray diffraction (XRD) pattern, transmission electron microscopy (TEM) images and vibrating sample magnetometer (VSM) curve. The antibacterial activities are investigated against* S. aureus* and *E. coli* as the Gram-positive and Gram-negative bacteria, respectively. The UV–Vis absorption was also studied in the present of NMMNPs at different time intervals that disclosed decreasing of the bacterial concentration. More than 80% of the bacteria were disappeared after treating in the presence of NMMNPs for 18 h. The Ni-NPs revealed an excellent mirror attribute with a well-controlled transmission (7%). A better light-reflectivity over conventional glass or a mercury mirror proved their utility for domestic uses in comparison with conventional mirrors as rather toxic materials like mercury. Owing to its magnetic properties, this kind of mirror can be easily made onto glass by using an external magnet. An ordered crystalline structure, admissible magnetic properties, substantial antibacterial activities, tunable mirror properties, mild reaction conditions, and overall, the facile synthesis are the specific features of the present protocol for the possible uses of NMMNPs in diverse applications.

## Introduction

Nanomaterials (NMs) in general render surface-controlled properties owing to a large surface-to-volume ratio, active energy carriers, and other variants useful for a variety of domestic uses^1–10^. Surface spins play a vital role in finely tuning magnetism and magnetic properties in magnetic nanoparticles (MNPs) of transition metals and other materials^11–16^. Several metals, such as Ag, Co, Ni, and Pd have been studied in the forms of NPs, with unique traits that promoted their usages in different applications^17–20^. Due, to remarkable properties like high reactivity, operational simplicity, biocompatibility, bacterial resistance, cost-effectiveness, abundance, anti-inflammatory activities, and environmental compatibilities, Ni-NPs attracted enormous interests and applied as a catalyst, an electro-catalyst, a photo-catalyst, a biosensor, and a heat-exchanger^21–40^. Many methods have been employed for the synthesis of Ni-NPs, namely, chemical reduction processes, discharge route, photocatalytic reduction, etc^41–44^. Most of these methods suffer from several disadvantages such as complex processing, complex reaction conditions, high temperatures, and long reaction times.

**Figure 1:**
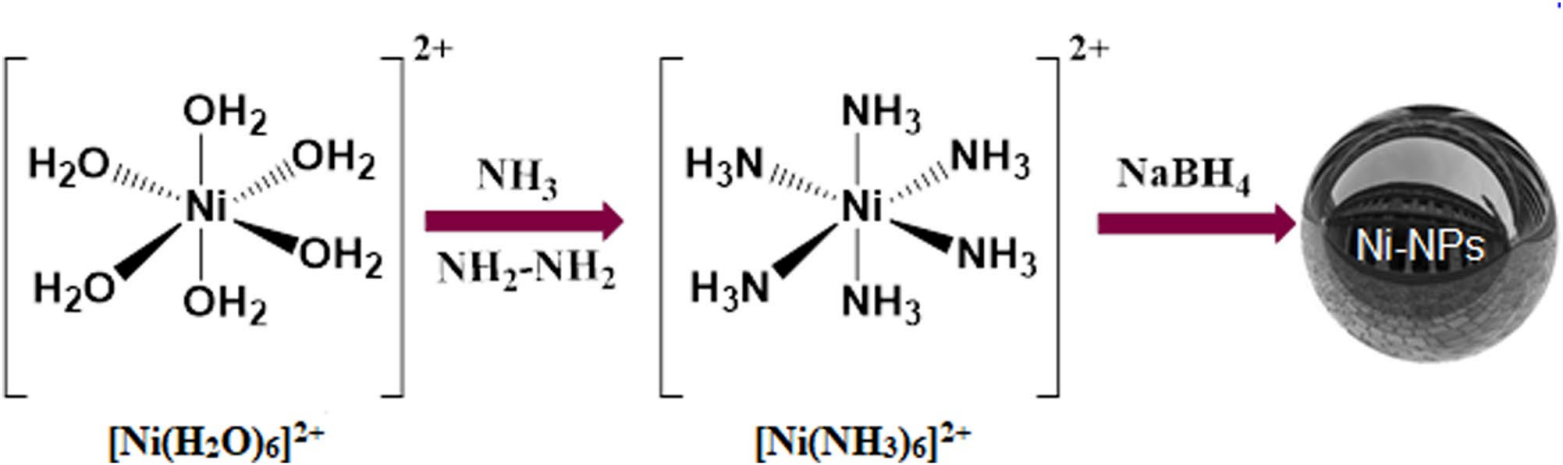
A schematic view of preparing NMMNPs in this work.

Synthesis of mirror films with NPs such as mercury, silver and gold has been reported in the various processes^45–47^. Most of the reported methods use toxic and expensive materials in which the mirror forms under challenging conditions. Significant characteristics of the mirror are its long-term durability and reflectivity, which are usually used in optical activities, wave-fronts, and solar cells^48–54^. It is an assembly of NPs that determines its reflectivity in the ultraviolet (UV)-visible regions of the electromagnetic spectrum^55,56^. Obviously, it is essential to prevent the risk of harmful bacteria in a public-health-safety. Therefore, various kinds of antibacterial agents are being developed. On the other hand, undesirable effects and consecutive of them lead to resistant to antibiotics. So, the synthesis of biocompatible antibacterial agents is one of the prime issues for scientists. The use of suitable NPs provides a high specific surface area and a high number density of active sites over the bulk values for these activities^6,57–61^. The metals like Ag, Ni, Zn, etc. are usefully reactive with proteins, and can stop the regulated transport across the plasma membrane by permeability affecting the transport system and lead to the death of bacteria^62–65^. Some NMs, including Ag, Cu, or ZnO, act strongly against the vast species of bacterial and fungal^66–69^. Hence, the introduction of NMs with the features mentioned above by a green synthetic route is highly demanded. In this regard, due to the importance of mirrors in different industries like space telescopes, optoelectronics, and medicals, the design and synthesis of magnetic mirrors with fewer toxicity materials is highly appreciated^70–74^. Also, the mirrors with antibacterial properties are required in medical instruments. Therefore, in this work, nickel magnetic mirror nanoparticles (NMMNPs) are designed, synthesized and characterized via efficient procedures, showing excellent magnetic properties, antibacterial activities and diverse mirror properties (Fig. 1). The antibacterial activities are studied against *S. aureus*, a Gram-positive bacterium, and *E. coli*, a Gram-negative bacterium. The synergic properties of these NMMNPs can be used safely for various industrial applications.

## Results and discussion

In this research, Ni-MNPs with mirror properties are synthesized for the first time by a simple method at ambient temperature in the water as an eco-friendly solvent. The synthesized NMMNs are characterized in terms of the FESEM/TEM images, XRD pattern, EDX spectrum, and magnetic properties. Ni retrievable MNPs are applied as a perfect mirror and the transmission is checked by their UV absorption spectra. Furthermore, acceptable antibacterial activities are studied by an agar disk diffusion and optical density (OD) analysis as follows.

### Characterization of NMMNPs

**Figure 2:**
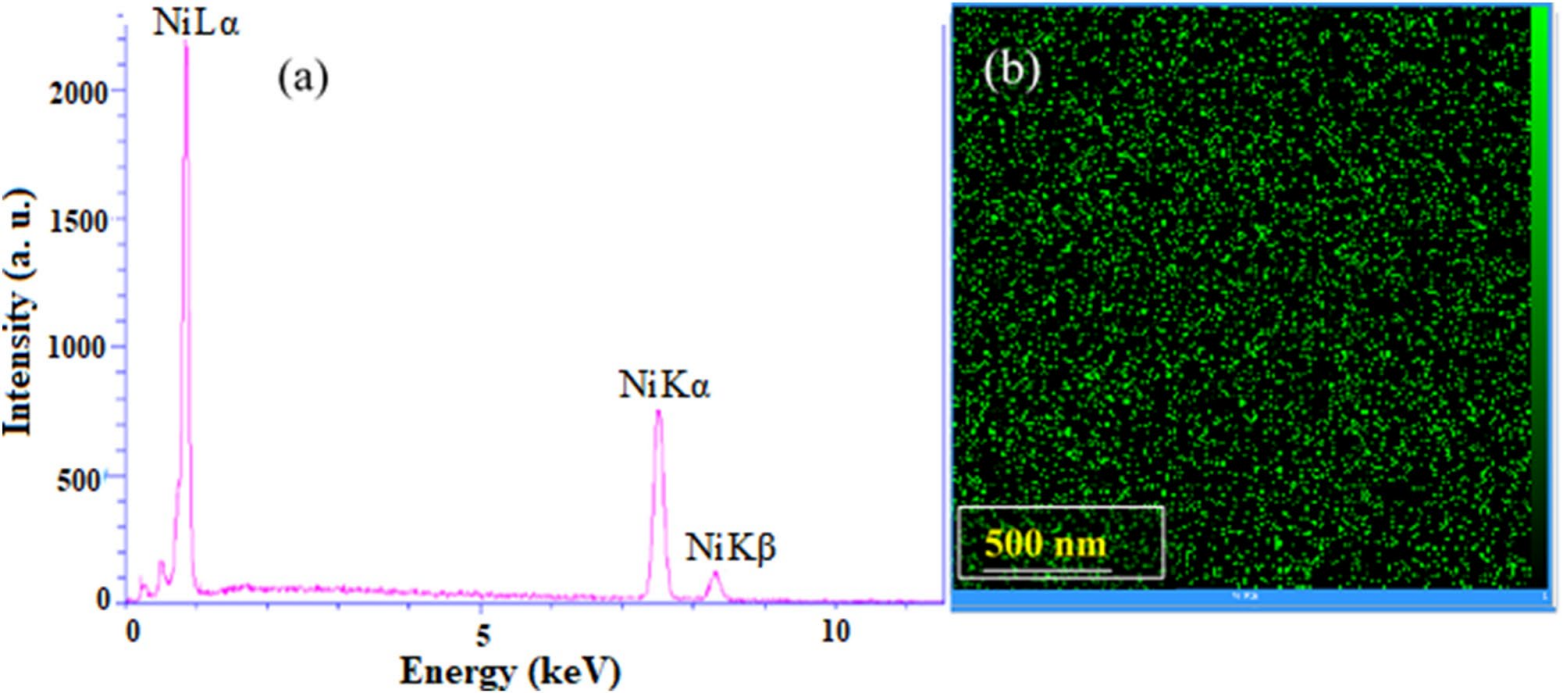
**(a)** EDX and **(b)** elemental mapping spectra of NMMNPs.

**Figure 3:**
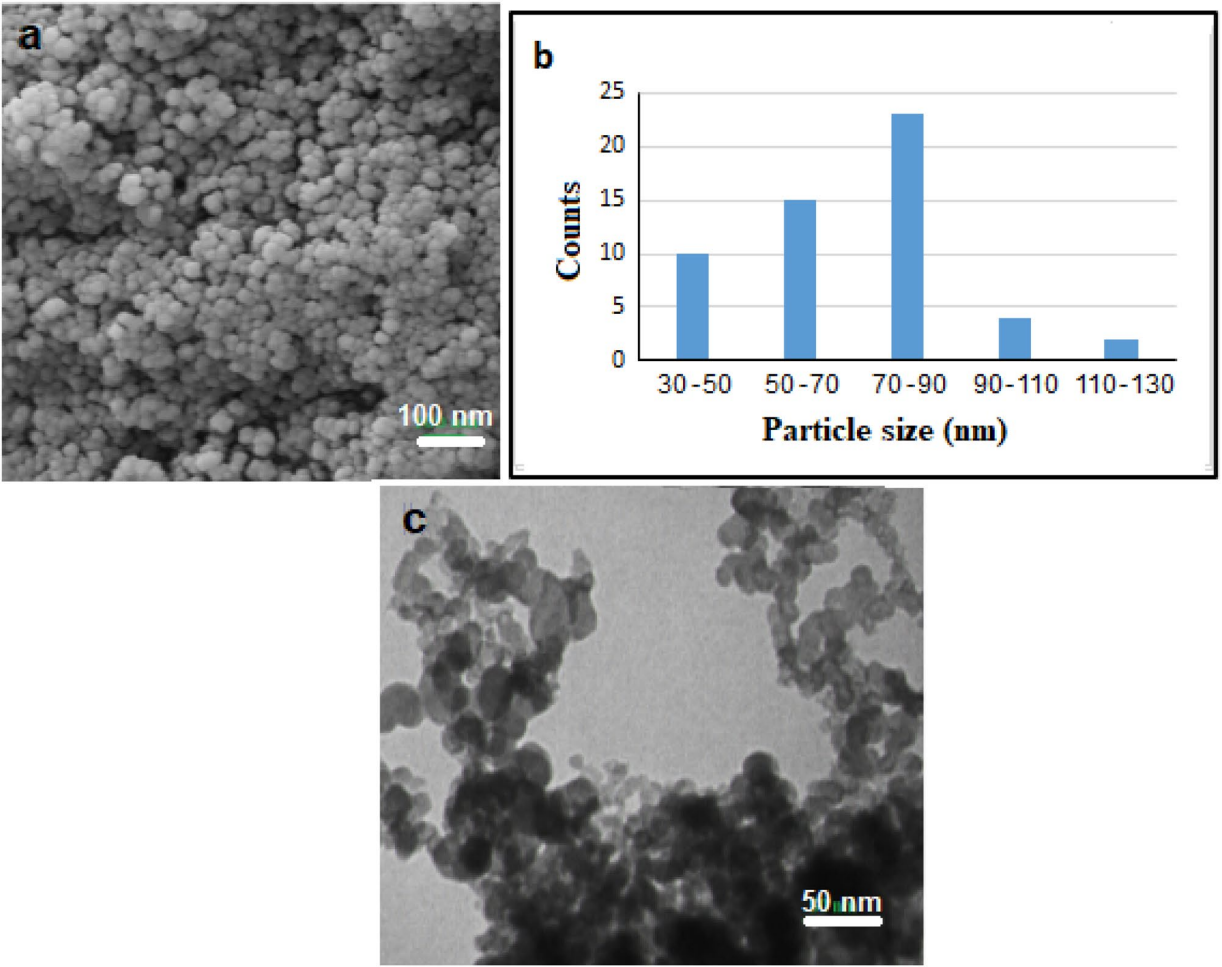
**(a)** FESEM images, **(b)** corresponding size distribution, and **(c)** TEM images of a sample of NMMNPs.

An EDX analysis is performed to ensure the nickel content in the NMMNs. As can be seen in a typical EDX spectrum in Fig. 2a, a pure Ni is present in a single phase with a practically uniform distribution in the elemental mapping in Fig. 2b. The purity of the Ni-NPs was checked by ICP analysis, in which the Ni-NPs were dissolved in concentrated nitric acid and kept for 3 days to make a clear solution. Some residual boron (from NaBH_4_ used in the reaction) was found below 10%. Figure 3a shows a reasonably uniform distribution of FESEM images of NMMNs at a scale of 30 to 130 nm. Their size distributions are portrayed in a bar diagram in Fig. 3b in a majority of NPs have 50 to 90 sizes. A closer view of TEM images in Fig. 3c reveals mostly rectangular prisms of bit smaller sizes of the NMMNPs crystallites, mostly are varied in a 20 to 40 nm range. In fact, the small crystallites are arranged further in clusters of a hierarchical structure as observed in duly bigger sizes in the FESEM images in Fig. 3a.

**Figure 4:**
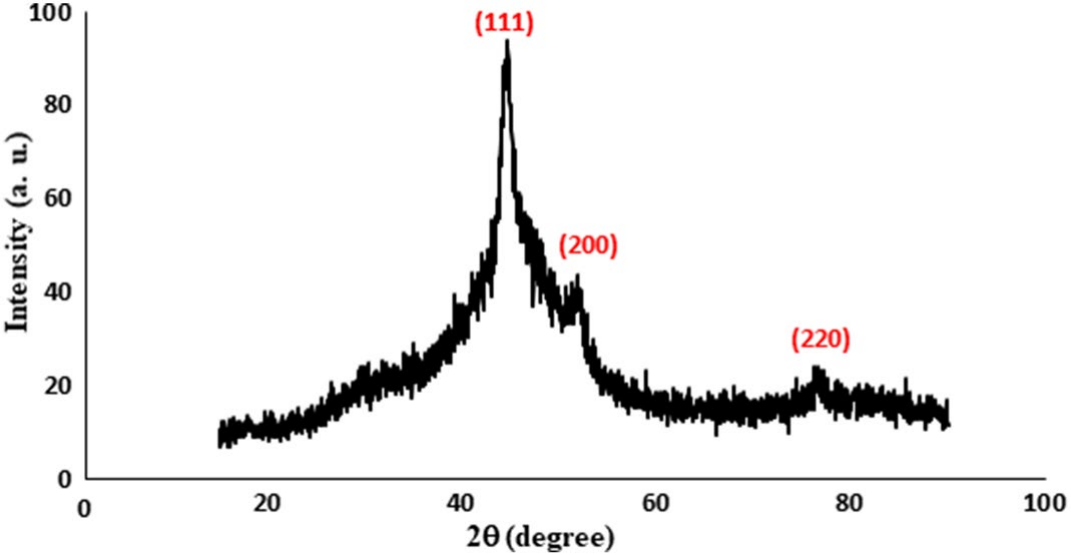
XRD pattern of a sample of NMMNPs.

Figure 4 shows the XRD pattern of three broad peaks of the sample of NMMNPs at diffraction angles 44.49°, 51.85° and 76.38° in the lattice reflections from the (111), (200), and (220) lattice planes, respectively, with an average lattice parameter *a* = 0.3522 nm of a well-known fcc structure. A bit larger *a*-value is observed in comparison to a bulk fcc-Ni of *a* = 0.3517 nm of JCPDS card no. 01-087-0712. An average crystallite size D = 30 nm is calculated using the peak broadening in the Scherrer relation as described elsewhere^32,33^.

**Figure 5:**
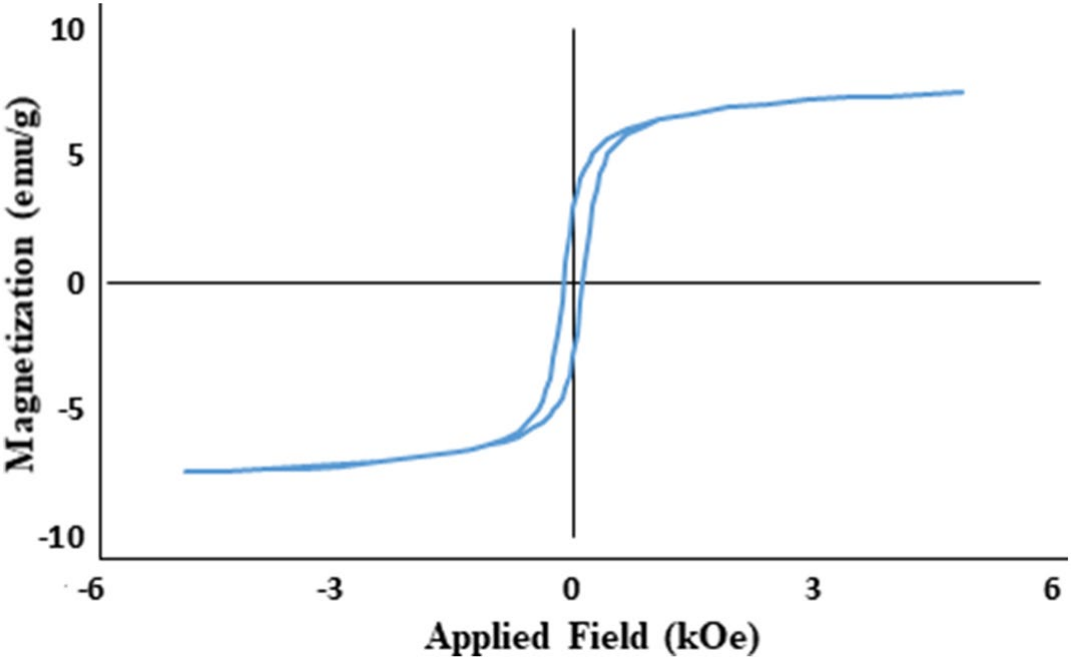
Magnetic hysteresis loop of a sample of NMMNPs.

A sample of NMMNPs exhibits a closed M-H hysteresis loop of magnetization (M) in Fig. 5 as measured in an applied magnetic field H of (-) 6 kOe to ( +) 6 kOe at room temperature. The loop is well symmetrical of its shape over the field H, with a coercivity H_c_ = 110 Oe and an effectively lower saturation magnetization M_s_ = 12 emu/g, intrinsic of a soft ferromagnetic phase. A reduced M_s_ = 12 emu/g value (over a nearly 54.5 emu/g in a pure fcc-Ni)^23,26,27^ observed here accounts in surface effects of the small crystallites of a cluster. A consistently small M_s_ is reported in Ni-NPs to form a soft magnetic mirror^42,76,77^.

### Antibacterial properties of NMMNPs

**Figure 6:**
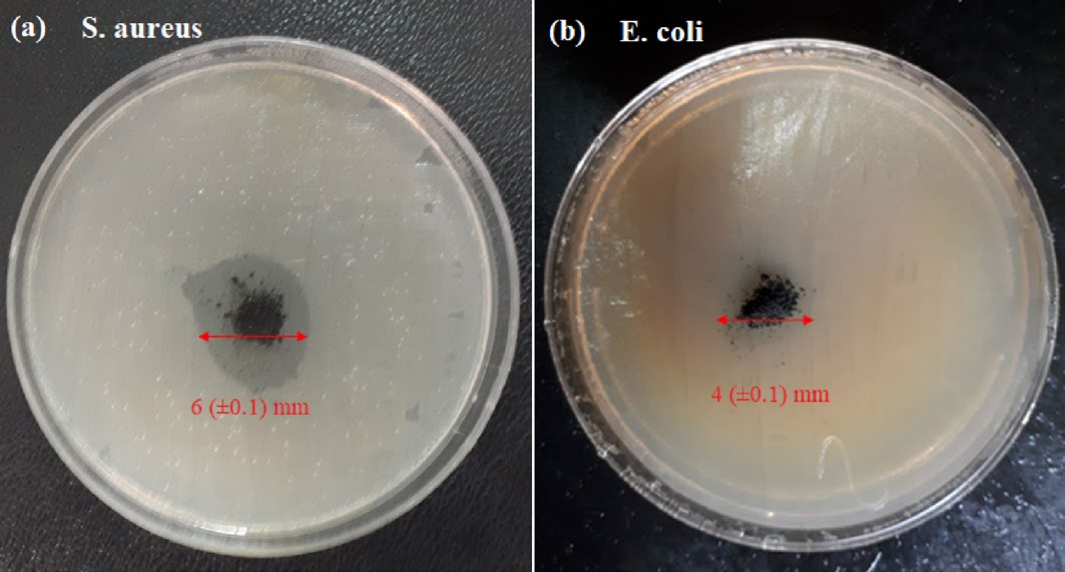
Agar disk diffusions of **(a)** *S. aureus* and **(b)** *E. coli* in the presence of NMMNPs.

Figure 6 presents the digital images of Agar disk diffusions of (a) *S. aureus* and (b) *E. coli* in the presence of the NMMNPs developed in this work. As can be seen in the presence of 0.01 g Ni-NPs at 37 °C for 24 h, there is no any visible bacterial growth (zone) around 0.6 and 0.4 cm for *S. aureus* and *E. coli*, respectively, in the present experiments.

**Figure 7:**
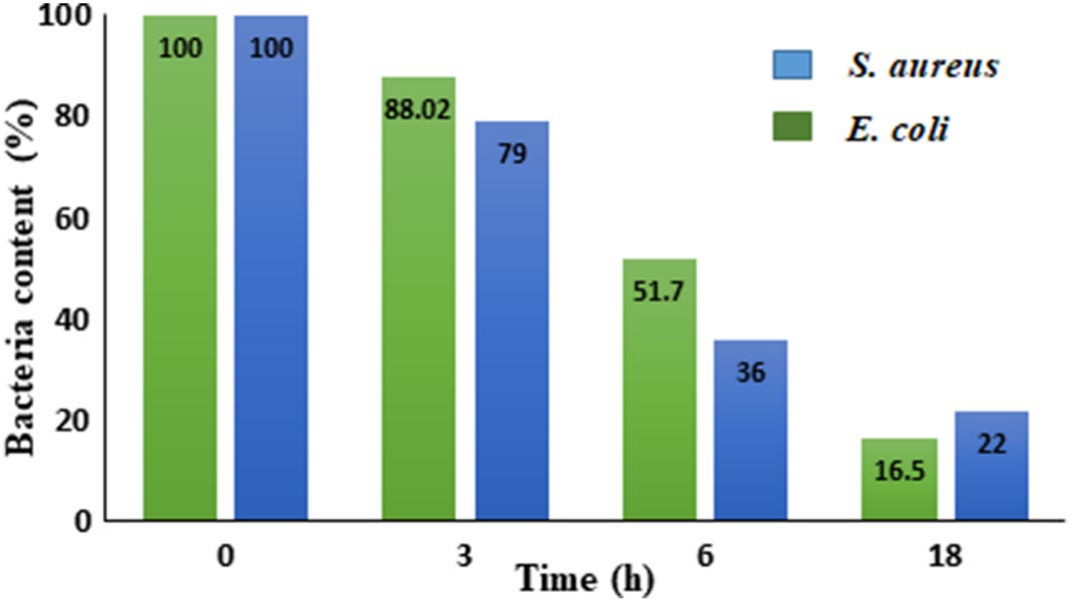
The OD diagram of *S. aureus* and *E. coli* in the presence of NMMNPs.

Further, we studied the OD measurements of bacterial cultures in the presence of 0.01 g Ni-NPs, 0.5 McFarland turbidity standard, and nutrient Broth media. The rate growth of bacterial was studied in 3, 6 and 18 h of stipulated time-intervals. As can be seen in a bar diagram in Fig. 7. The antibacterial activities show a noticeable inhibition of bacterial growth for *E. coli* compared to *S. aureus* after 6 h. However, only less than 22% of the bacteria remained viable after 18 h in both cases. The results confirm the effect of NMMNPs killing and inhibiting bacterial growth in terms of the UV absorption spectra in a SI file.

**Figure 8:**
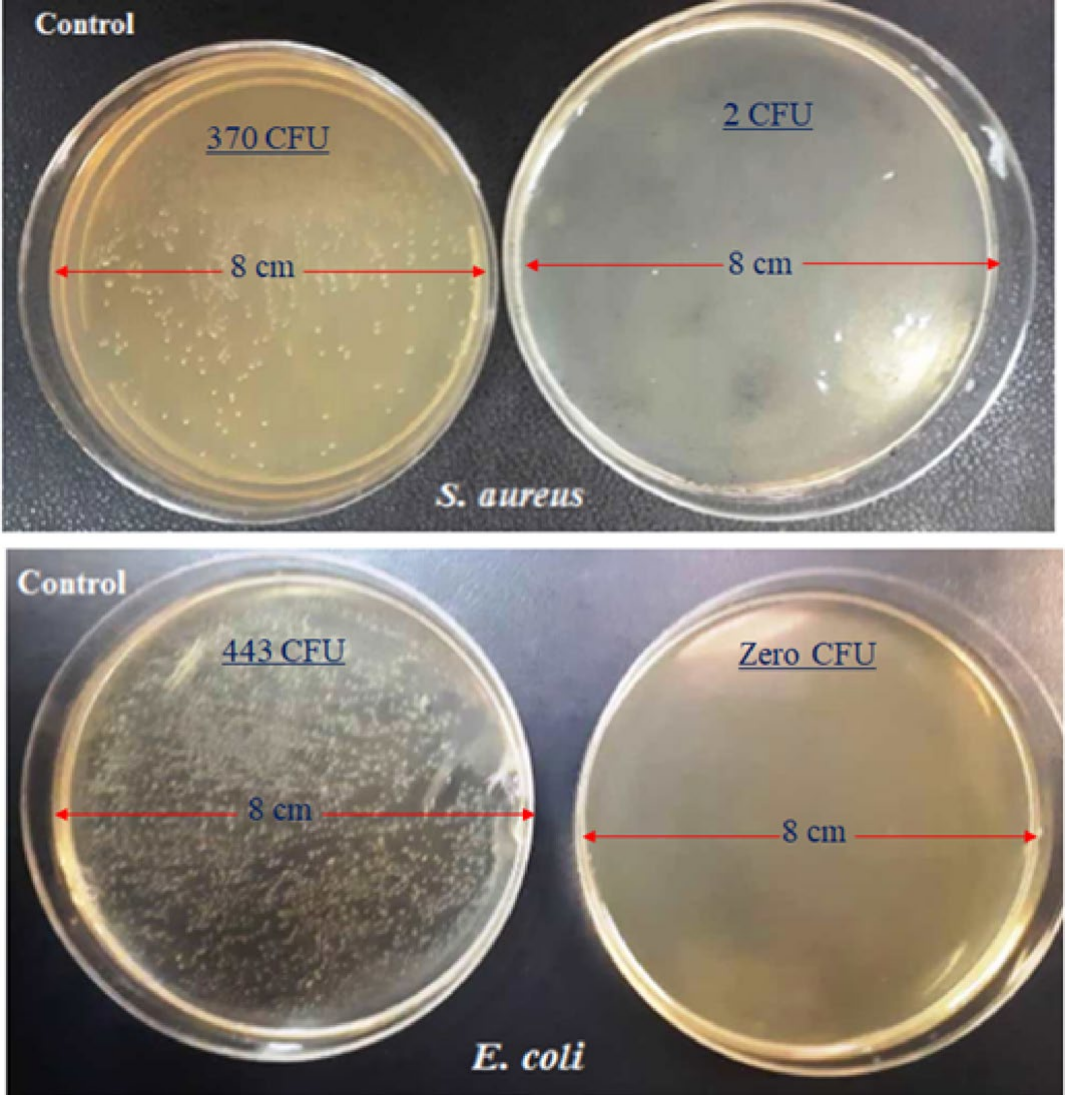
Images of *S. aureus* and *E. coli* in the absence and presence (in the right) of NMMNPs after 24 h of stipulated exposures.

As usual, the colony counter method was applied to estimate the concentration of live bacteria in the cultured samples^78–82^. From the digital images given in Fig. 8, it is clear that the NMMNPs had inactivated the *E. coli* and *S. aureus* bacteria upon a critical 24 h of exposure. Similarly, the antibacterial performance of the Ni-NPs was evaluated in comparison to the results of the control obtained in the absence of the Ni-NPs under identical conditions.

### Antimicrobial activities of NMMNPs

NMMNPs have antibacterial properties on *E. coli* and *S. aureus* in the bacterial plasma membrane is changed as comes in contact to Ni-NPs^78,79^. Cell death incurs by its permeability affecting its proper transport through the plasma membrane, leaving the bacterial cells incapable of properly regulating the due transport through the plasma membrane. Also, Ni-NPs have penetrated inside the bacteria and believed to damage them by interacting with phosphorous and sulfur containing compounds such as DNA. On exposed to selected bacterial, NMMNPs can release Ni ions and show the bactericidal efficiency^82,83^.

### Mirror properties of NMMNPs

**Figure 9:**
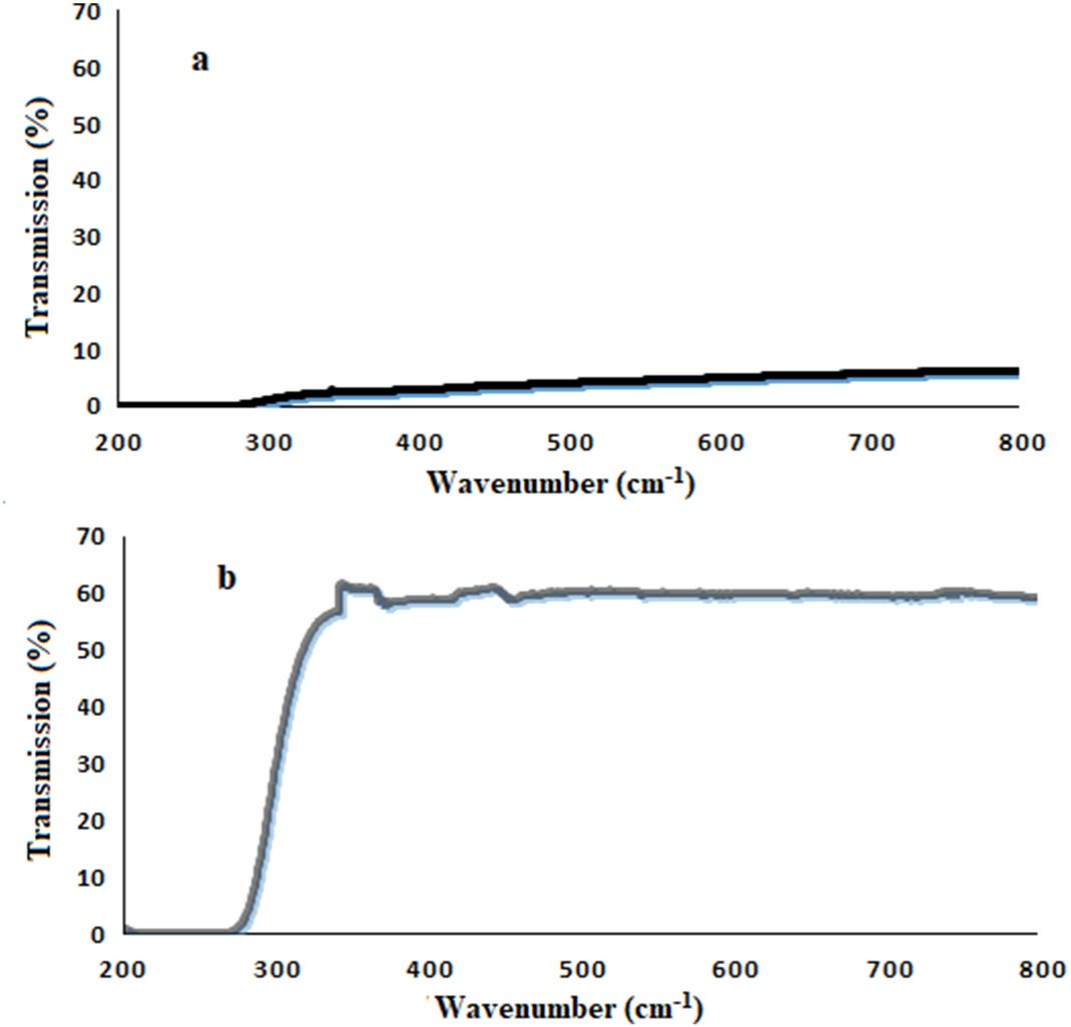
Transmittance spectra of **(a)** NMMNPs and **(b)** glass.

The mirror property of the Ni-NPs is confirmed in terms of the fractional values of transmittance and reflectance of the sample. A wide UV–visible region of 300 to 800 nm was chosen for comparing the transmission values in the synthesized mirror and a reference glass. As shown in the spectra in Fig. 9, the two samples have the values of 7% and 70%, respectively. Evidently, Fig. 9a, the mirror passes only a small fraction of light in the UV–visible regions. Figure 9b, and glass transmits a lot of UV–visible regions.

**Figure 10:**
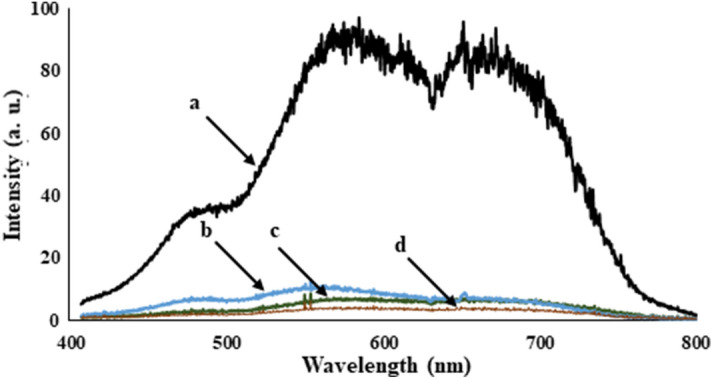
Intensity of light received to detector from **(a)** a tungsten halogen lamp, **(b)** a mercury mirror, **(c)** the synthesized NMMNPs, and **(d)** a routine glass.

**Figure 11:**
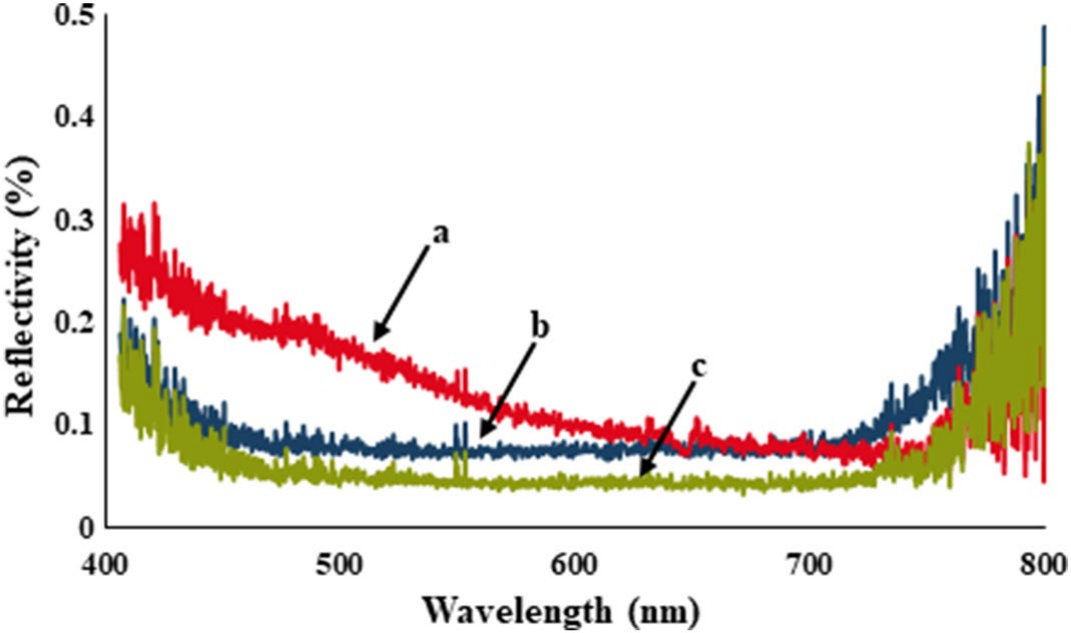
The reflectivity spectra of **(a)** a mercury mirror, **(b)** the synthesized NMMNPs, and **(c)** a routine glass.

Furthermore, the reflectance of NMMNPs was studied using a tungsten halogen lamp over in 340–850 nm range. A known intensity (*I*_1_) of the light source was used to obtain the reflected values (I_2_) from the mercury mirror, synthesized Ni-NPs, and a routine glass in terms of the reflectance spectra, as given in Fig. 10. Here, the mercury mirror displays a reasonably higher value than the glass and NMMNPs. A value of the reflectivity (%) = I_2_/I_1_ was estimated using the so obtained I_1_ and I_2_ values. As portrayed in Fig. 11, the mercury mirror has a better reflectance than the NMMNPs over an early 400–600 nm regime, while the NMMNPs resume a higher reflectance at the longer 700–800 nm wavelengths.

**Figure 12:**
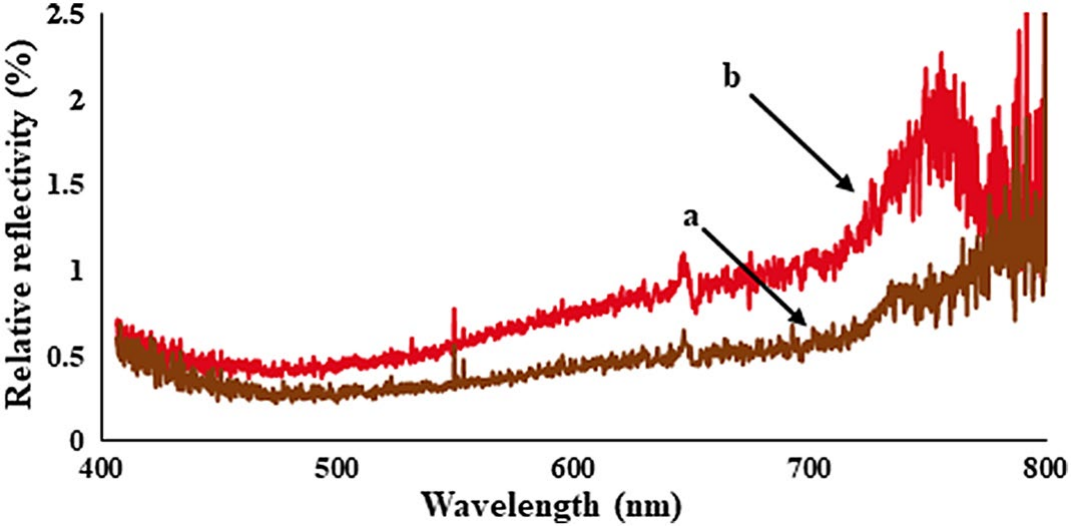
Reflectivity spectra of **(a)** glass and **(b)** NMMNPs over the mercury as a reference.

In general, the supremacy of the synthesized mirror of Ni-NPs can be visualized by expressing its relative reflectivity with respect to the mercury value. As this is portrayed in Fig. 12, the NMMNPs have quite acceptable value if compared to a routine glass what is it can be used as an alternative to the mirrors of toxic materials like mercury.

## Experimental

### Synthesis of NMMNPs

In a typical reaction batch, a 2 g of NiCl_2_·6H_2_O was taken in a 100 mL distilled water in a round-bottom flask to make a [Ni(H_2_O)_6_]^2+^ complex. To form an aqueous complex [Ni(NH_3_)_6_]^2+^, solutions of NH_3_ and hydrazine hydrate (each of 20 mℓ) were added drop by drop to the green NiCl_2_·6H_2_O solution. The mixture was stirred for 2 h and then, a 0.20 g of NaBH_4_ was added after the black solution resident stays for 48 h. The mirror was created on the internal surface of the bottom flask. Then, the extra solution was removed from the bottom, and the Ni-NPs were separated by an external magnet. To antibacterial tests and other analyses, the NMMNPs were washed repeatedly by distilled water and dried at 70 °C in an ambient atmosphere.

### Structural analyses

The pure materials like metal salt, solvents and other chemicals were obtained from Sigma-Aldrich and Merck companies. The FESEM images were recorded by using a TESCAN VEGA3 microscope (with a provision to study EDX spectra from selected regions in conjunction with a Numerix DXP-X10P analyzer), while the TEM images were obtained by using a Philips CM120 instrument. The Bruker D8 advance was applied to measure the XRD patterns of the various samples. The magnetic properties were studied using a vibrating sample magnetometer (VSM) of Lakeshore 7407 (Meghnatis Kavir Kashan Co., Iran). A Shimadzu UV–visible Mini 1240 spectrophotometer was used to study the absorption spectra in the UV–visible region. The ICP analysis was provided on a Shimadzu ICPS-7000. An agar disk diffusion test was applied by agar plates that include an agar as a solid growth medium and nutrients microorganisms for antibacterial tests. Further, a 0.5 McFarland turbidity standard to Nutrient Broth media was applied for the antibacterial test. A tungsten halogen lamp was used to study the reflectance spectra in a 340–850 nm range.

### Procedure of the antibacterial test

Gram-negative *Escherichia coli* and *Staphylococcus aureus* as Gram-positive bacteria were applied for antibacterial tests. A 0.01 g of NMMNPs was added to the agar plate containing bacteria. The Petri dishes were kept at 37 °C in an incubator for 24 h. All of the instruments have been sterilized at 121 °C. Further, the OD measurements were done by the addition of a 0.01 g of NMMNPs with 0.5 McFarland turbidity standard to Nutrient Broth media. The rate of bacterial killing was checked at various time-intervals in terms of the UV–visible spectra of the recovered samples. For the colony counter, a diluted 0.5 McFarland turbidity standard, a 0.1 g of Ni-NPs and a 0.2 mℓ of DMSO were added to nutrient Broth culture media. The mixture was kept at 37 °C in an incubator for 2 h. Then, a 0.1 mℓ of this mixture was kept in Mueller Hinton agar^78–83^.

## Conclusions

In summary, a simple, green, and efficient method has been introduced using a nickel-metal salt as a precursor for synthesizing Ni-NPs with useful features of the magnetic mirror, antibacterial activities and magnetic properties. The antibacterial activities of NMMNPs are studied for *S. aureus* and *E. coli bacteria *by a cup-plate agar diffusion method and OD measurements. The inactivated *E. coli* and *S. aureus* bacteria in the presence of NMMNPs were confirmed by colony method. The NMMNPs showed a marked sensitivity against *S. aureus* and *E. coli *in the *S. aureus* reveals a higher zone of inhibition than *E. coli* bacterium. An OD analysis verified that the killing and inhibiting bacterial growth is better than 80% in an 18 h exposure. The synthesized mirror has less than 7% transmission in a UV–visible light usefully for its possible applications. The NMMNPs are better reflective than a routine glass. Because of its low toxicity, a sample Ni-NPs can be used as an alternative to mirrors with toxic materials like Hg. Moreover, due to magnetic properties, a coating of NMMNPs on a glass as a highlight mirror feature of this report can be simply provided by an external magnet. As a result, in the reported properties and the synergistic effect of these features, the NMMNPs can be applied in domestic usages such as dentistry, surgery, laser tools, photonics, and space telescopes.

## Supplementary information

Supplementary Information.
