# Author Correction: Synthesis of nickel nanoparticles by a green and convenient method as a magnetic mirror with antibacterial activities

**DOI:** 10.1038/s41598-020-76399-2

**Published:** 2020-10-30

**Authors:** Mohammad Reza Ahghari, Vahhab Soltaninejad, Ali Maleki

**Affiliations:** Catalysts and Organic Synthesis Research Laboratory, Department of Chemistry, Iran University of Science and Technology, Tehran, 16846‑13114 Iran

Correction to: *Scientific Reports* 10.1038/s41598-020-69679-4, published online 28 July 2020

The Article contains an error in the Reference list. The authors erroneously included the below paper, which is listed as Reference 77.

77. Rausch, P., Verpoort, S. & Wittrock, U. Unimorph deformable mirror for space telescopes: Environmental testing. *Opt. Express* **24**, 1528–1542 (2016).

There should be only references 42 and 76 cited in the Results and discussion section, where

“A consistently small M_s_ is reported in Ni-NPs to form a soft magnetic mirror 42, 76, 77.”

should read:

“A consistently small M_s_ is reported in Ni-NPs to form a soft magnetic mirror 42, 76.”

