# Supplementary material for: Synthesis of nickel nanoparticles by a green and convenient method as a magnetic mirror with antibacterial activities
Source: Sci Rep. 2020 Jul 28;10:12627. doi: 10.1038/s41598-020-69679-4 (PMC7387540; doi:10.1038/s41598-020-69679-4)
Supplement: Supplementary file 1 — Supplementary Information. [file 41598_2020_69679_MOESM1_ESM.pdf]

Synthesis of nickel nanoparticles by a green and convenient method as a magnetic mirror with antibacterial activities

Mohammad Reza Ahghari, Vahhab Soltaninejad and Ali Maleki*

Catalysts and Organic Synthesis Research Laboratory, Department of Chemistry, Iran University of Science and Technology, Tehran 16846-13114, Iran

**Corresponding author.* Fax: +98-21-73021584; Tel: +98-21-73228313;

E-mail: *maleki@iust.ac.ir*

Table of contents

Subject **Page**

Figure S1. UV spectrum of *E. coli* in the presence of NMMNPs after 3h …..…..…….……….………….S2

Figure S2. UV spectrum of *E. coli* in the presence of NMMNPs after 6h …………………….….……..S3

Figure S3. UV spectrum of *E. coli* in the presence of NMMNPs after 18h …………..…………..…...…...S4

Figure S4. UV spectrum of *S. aureus* in the presence of NMMNPs after 3h ……………………….……S5

Figure S5. UV spectrum of *S. aureus* in the presence of NMMNPs after 6h ……...…………..…….……..S6

Figure S6. UV spectrum of *S. aureus* in the presence of NMMNPs after 18h …………………..…...…..S7

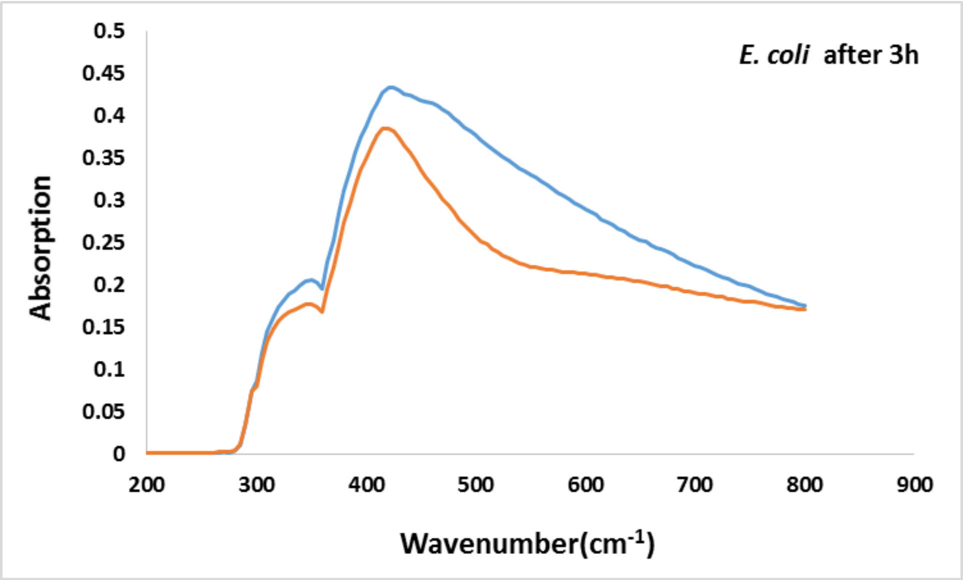

Figure S1. UV spectrum of *E. coli* in the presence of NMMNPs after 3h.

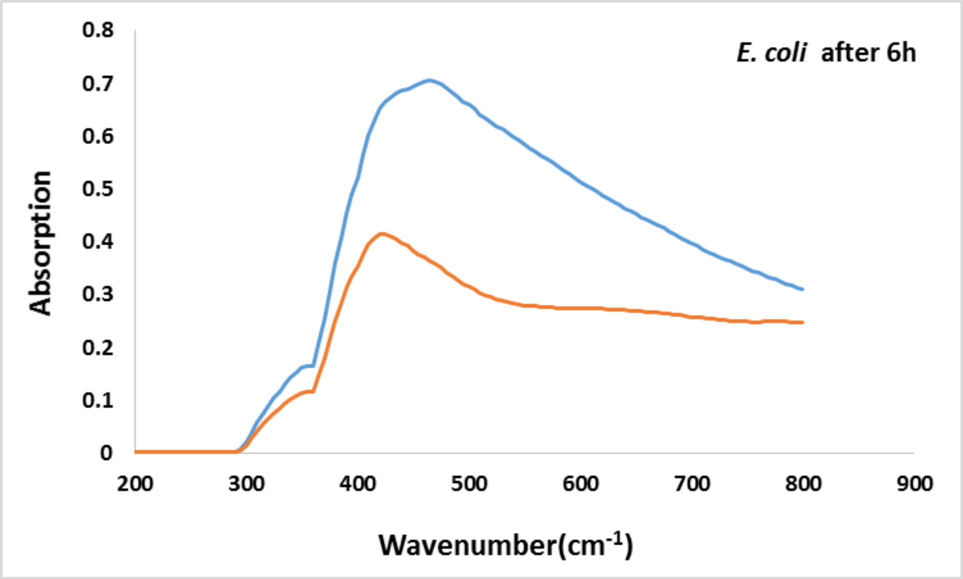

Figure S2. UV spectrum of *E. coli* in the presence of NMMNPs after 6h.

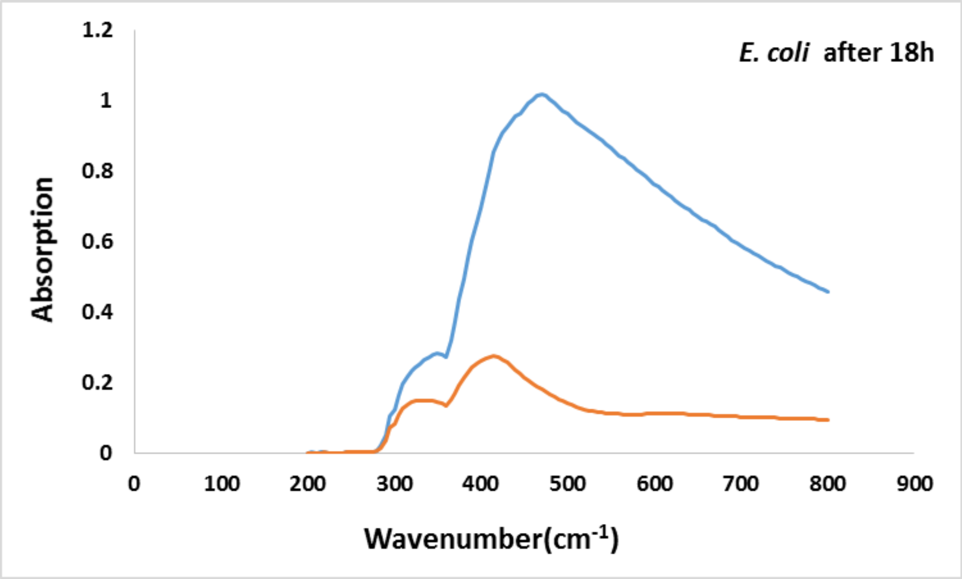

Figure S3. UV spectrum of *E. coli* in the presence of NMMNPs after 18h.

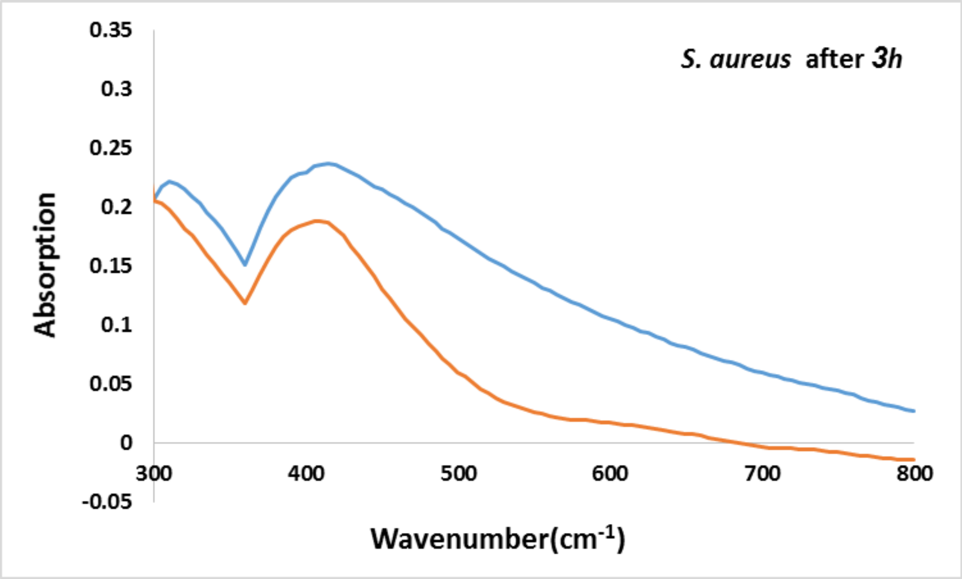

Figure S4. UV spectrum of *S. aureus* in the presence of NMMNPs after 3h.

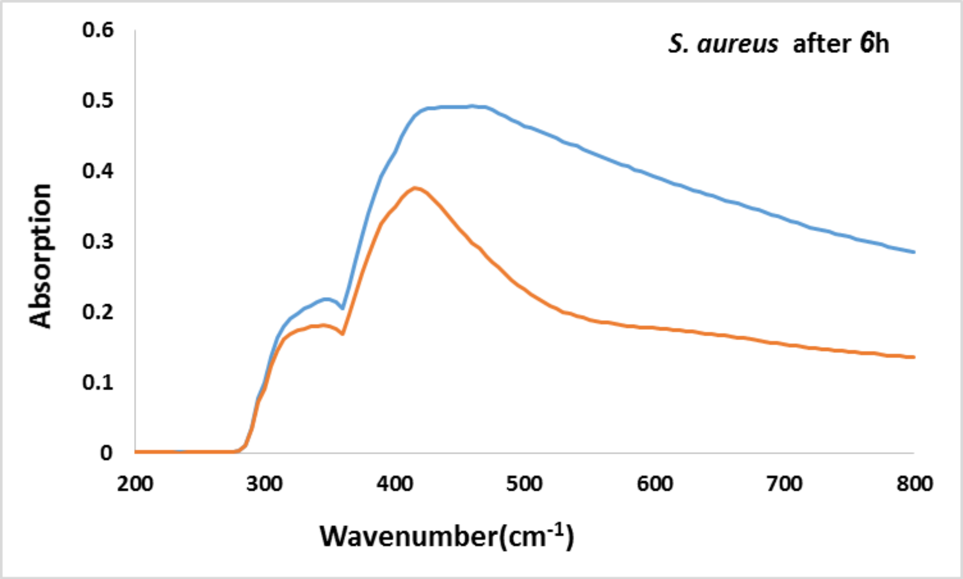

Figure S5. UV spectrum of *S. aureus* in the presence of NMMNPs after 6h.

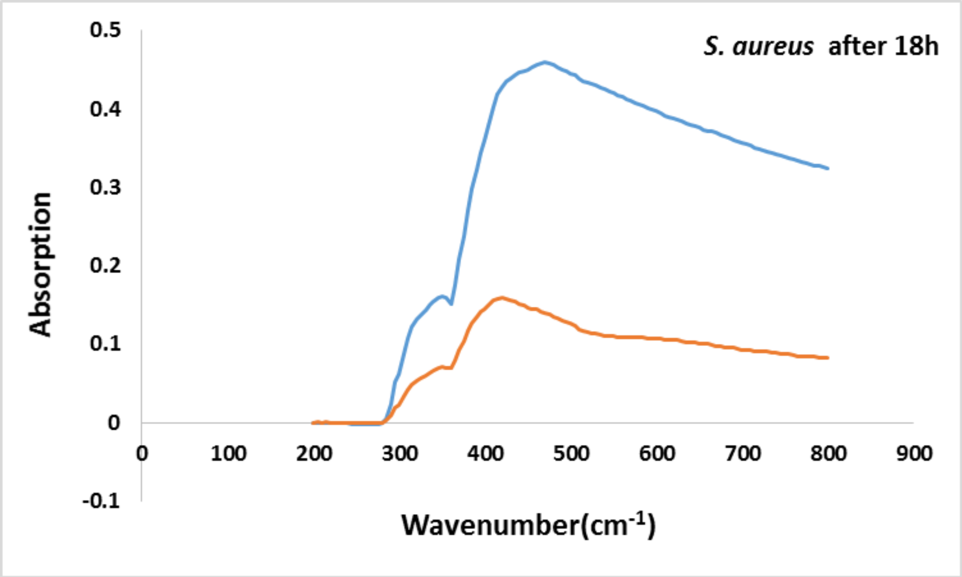

Figure S6. UV spectrum of *S. aureus* in the presence of NMMNPs after 18h.
